# Changes in Metabolic Activity and Gait Function by Dual-Task Cognitive Game-Based Treadmill System in Parkinson’s Disease: Protocol of a Randomized Controlled Trial

**DOI:** 10.3389/fnagi.2021.680270

**Published:** 2021-06-04

**Authors:** Tony Szturm, Tiffany A. Kolesar, Bhuvan Mahana, Andrew L. Goertzen, Douglas E. Hobson, Jonathan J. Marotta, Antonio P. Strafella, Ji Hyun Ko

**Affiliations:** ^1^College of Rehabilitation Sciences, University of Manitoba, Winnipeg, MB, Canada; ^2^Department of Human Anatomy and Cell Science, University of Manitoba, Winnipeg, MB, Canada; ^3^Department of Radiology, University of Manitoba, Winnipeg, MB, Canada; ^4^Department of Internal Medicine, University of Manitoba, Winnipeg, MB, Canada; ^5^Department of Psychology, University of Manitoba, Winnipeg, MB, Canada; ^6^Morton and Gloria Shulman Movement Disorder Unit, E. J. Safra Parkinson Disease Program, Neurology Division/Department of Medicine, Toronto Western Hospital, Krembil Brain Institute, University Health Network (UHN), Brain Health Imaging Centre, Campbell Family Mental Health Research Institute, CAMH, University of Toronto, Toronto, ON, Canada

**Keywords:** Parkinson’s disease, dual-task, randomized controlled trial, positron emission tomography, magnetic resonance imaging

## Abstract

Balance and gait impairments, and consequently, mobility restrictions and falls are common in Parkinson’s disease (PD). Various cognitive deficits are also common in PD and are associated with increased fall risk. These mobility and cognitive deficits are limiting factors in a person’s health, ability to perform activities of daily living, and overall quality of life. Community ambulation involves many dual-task (DT) conditions that require processing of several cognitive tasks while managing or reacting to sudden or unexpected balance challenges. DT training programs that can simultaneously target balance, gait, visuomotor, and cognitive functions are important to consider in rehabilitation and promotion of healthy active lives. In the proposed multi-center, randomized controlled trial (RCT), novel behavioral positron emission tomography (PET) brain imaging methods are used to evaluate the molecular basis and neural underpinnings of: (a) the decline of mobility function in PD, specifically, balance, gait, visuomotor, and cognitive function, and (b) the effects of an engaging, game-based DT treadmill walking program on mobility and cognitive functions. Both the interactive cognitive game tasks and treadmill walking require continuous visual attention, and share spatial processing functions, notably to minimize any balance disturbance or gait deviation/stumble. The ability to “walk and talk” normally includes activation of specific regions of the prefrontal cortex (PFC) and the basal ganglia (site of degeneration in PD). The PET imaging analysis and comparison with healthy age-matched controls will allow us to identify areas of abnormal, reduced activity levels, as well as areas of excessive activity (increased attentional resources) during DT-walking. We will then be able to identify areas of brain plasticity associated with improvements in mobility functions (balance, gait, and cognition) after intervention. We expect the gait-cognitive training effect to involve re-organization of PFC activity among other, yet to be identified brain regions. The DT mobility-training platform and behavioral PET brain imaging methods are directly applicable to other diseases that affect gait and cognition, e.g., cognitive vascular impairment, Alzheimer’s disease, as well as in aging.

## Introduction

Disruption of frontostriatal circuits in Parkinson’s disease (PD) have been shown to be associated with gait impairments (Rochester et al., 2004; Rosenberg-Katz et al., 2015) and deficits in executive cognitive functions (Kudlicka et al., 2011; Olchik et al., 2017). It is now well established that gait and cognition are closely linked (Plotnik et al., 2011; Kelly et al., 2012; Callisaya et al., 2016). For example, community ambulation requires walking while also performing a variety of executive cognitive functions, such as navigating busy environments, managing complex terrains, searching for and tracking various visual targets, information processing of what is being seen, reading, etc. Unfortunately, both mobility skills and cognition are often affected in PD (Mak et al., 2014; Paul et al., 2014; Schneider et al., 2015).

The dual-task (DT) paradigm has been used in a number of studies to assess the magnitude of DT interference of cognitive load on gait function and vice versa in PD (Rochester et al., 2014; Heinzel et al., 2016; Penko et al., 2018). DT testing is considered representative of real-life situations, and provides better insight into functional capacity. The concept of motor automaticity has an important implication in the pathophysiology of PD related to mobility limitations and increased fall risk (Wu et al., 2015; Gilat et al., 2017). One model of executive cognitive functions presents the supervisory attentional system, which distinguishes between processing of non-routine, attentionally demanding activities vs. routine, automated tasks (Dirnberger and Jahanshahi, 2013). The role of the sensorimotor striatum has been equated with that of routine tasks, such as locomotion, that are usually performed automatically (Gilat et al., 2017). The increased attentional and information processing demands of DT-walking are presumed to reduce the already limited locomotor control of PD patients (Kang et al., 2005; Wu and Hallett, 2008), and this may result in greater gait variability/instability and a higher risk of falls.

Emerging evidence suggests that the decline in mobility function due to a combination of motor and cognitive impairment can be improved by DT training (Gregory et al., 2017). For example, virtual environments viewed during treadmill walking provides a task-orientated approach to DT-walking training (Mirelman et al., 2016). Maximizing participation is also a main goal of any exercise program. Long-term exercise programs are often fraught with low compliance and adherence, so maintaining motivation is thus central to long-term functional success of a therapy program. A promising methodology is to combine exercise with computer games, making training a more engaging and enjoyable experience (Barry et al., 2014). Digital media in the form of computer games can also challenge and improve many different aspects of executive cognitive function (Strenziok et al., 2014; Bonnechère et al., 2020). Many common and modern computer games are readily available in which processing speed, cognitive inhibition, task switching, working memory, and problem-solving are main components of interactive game play.

There is a need to develop and validate a low-cost platform that combines walking and executive cognitive activities. For this purpose, our research group has developed and validated an engaging cognitive game-based treadmill platform (GTP) for DT training, which includes an automated assessment subsystem (Nankar et al., 2017; Szturm et al., 2017; Ahmadi et al., 2019; Mahana et al., 2021). The GTP provides an integrated approach to address the decline in balance, gait, visuomotor, and executive cognitive functions. The GTP consists of: (a) a standard treadmill instrumented with a pressure mapping system to record center-of-foot pressure during walking, (b) an interactive computer game subsystem for DT; and (c) an automated monitoring application and analysis methods to quantify both gait and cognitive outcome measures (electronic records) while DT-walking.

At present, understanding of the neural underpinnings of DT-walking and training effects in PD is limited due to methodological limitations in neuroimaging (Monchi et al., 2004; Holtzer et al., 2014; Maidan et al., 2016). While diffusion tensor imaging (DTI) suggests that reduced white matter integrity—in pathways such as the forceps minor, corpus callosum, uncinate fasciculus, and cingulum—is associated with decreased gait stability and can indicate an increased risk of falling (Ghanavati et al., 2018; Snir et al., 2019), most neuroimaging data are limited to correlational analyses that take place in the absence of a walking task, or are limited in terms of spatial extent, such as functional near infrared spectroscopy (fNIRS). Some fNIRS investigations of walking have revealed that in healthy, able-bodied adults, prefrontal cortex (PFC) is bilaterally active and remains active during DT-walking as compared to single-task walking, whereas activation of the frontal lobe during DT-walking is impaired in persons with PD (Maidan et al., 2016). Recent functional magnetic resonance imaging (fMRI) studies have shown similar lack of PFC engagement during an executive function task (i.e., Wisconsin Card Sorting Task) in PD patients (Monchi et al., 2001; Nagano-Saito et al., 2014). It was suggested that basal ganglia degeneration affecting the striatum would result in less PFC activation during set-shifting in PD patients compared to the age-matched controls (Monchi et al., 2001).

Studies using resting fluorodeoxyglucose (FDG) positron emission tomography (PET) imaging have identified altered metabolic patterns in PFC and motor planning regions in PD that are associated with various gait abnormalities (and executive cognitive dysfunction (Fasano et al., 2015; Olchik et al., 2017). A few studies have examined FDG-PET in which the FDG uptake period involves either treadmill walking or overground walking (Shimada et al., 2013; Hinton et al., 2019; Mitchell et al., 2019a,b). FDG-PET imaging provides the unique opportunity to study functional imaging during interactions with different environments, in particular, during walking, because participants do not need to be constrained during the FDG uptake period. For example, PET imaging studies have been able to demonstrate a decline in sensorimotor cortical processing in middle-age during walking and obstacle negotiation (Mitchell et al., 2019b), and in PD patients with freezing of gait (Mitchell et al., 2019a). However, how DT-walking changes brain metabolism in PD has not been investigated. In this study, we will investigate behavioral analyses of gait and cognitive function, in addition to FDG-PET to assess a 10-week DT-walking training intervention, compared to treadmill walking only.

Although it is not possible to directly observe changes in brain function during a DT-walking task using MRI, this methodology can still provide important information regarding changes in brain structure and function at rest that can result following DT-walking training. Thus, in addition to the task-based PET imaging, pilot DTI and resting state data will be acquired—i.e., measuring brain function in the absence of an overt task—using both resting state fMRI (RS-fMRI) and pseudo continuous arterial spin labeling (pCASL). Although not much research has directly compared the relationship between gait impairment and functional connectivity—i.e., how different brain regions’ activity correlates over time—data suggest that PD patients with postural instability and gait difficulty have reduced functional connectivity in the cerebellum and putamen, compared to healthy controls (Chen et al., 2015; Ma et al., 2017; Tessitore et al., 2019). Furthermore, postural instability negatively correlates with functional connectivity in the putamen (Chen et al., 2015). Finally, using support vector machine-based [SVM, using an iterative single data algorithm (ISDA)] scores, our previous work shows that PD patients have similar metabolic and perfusion patterns (i.e., SVM-ISDA scores) as those with Alzheimer’s disease—importantly, these SVM-ISDA scores may be useful for monitoring disease progression and treatment response, and will be piloted in this study as a result (Katako et al., 2018).

## Material and Equipment

### DT Equipment

Both single-task (treadmill walking only) and DT conditions will use a standard treadmill instrumented with a pressure mapping system to measure center-of-foot pressure (Vista Medical Ltd., Winnipeg, MB, Canada). An interactive computer game system for DT will also be used, including a computer monitor for visual display (placed at the front of the treadmill), and a pool of 30 commercial games available from www.bigfishgames.com for the DT training program (see Supplementary Table 1 for a list and description of the games). Additionally, a miniature, wireless plug-n-play inertial-based computer mouse (IB-mouse; Therapy Mouse Mobility Research, AZ, United States) will be secured to a plastic headband and will allow interaction with the videogame system *via* head pointing movements (head rotation) to control the motion of any game sprite/paddle.

### Neuroimaging Equipment

Positron emission tomography images will be acquired using a Siemens Biograph mCT PET/CT scanner (Siemens Healthineers, Knoxville, TN, United States). Participants will be administered 185 MBq of [^18^F] FDG. For participants recruited in Winnipeg, MRI images will be acquired on a 3-Tesla Siemens/IMRIS MAGNETOM Verio MRI scanner (Erlangen, Germany), using a 12-channel head and neck coil. Participants recruited in Toronto will be scanned using a 3-Tesla, GE Discovery MR750 scanner.

## Methods

### Objectives

The aim of the proposed study is twofold. Behavioral analysis of gait and cognitive function together with PET and MRI brain imaging methods will be used to evaluate the molecular basis and neural underpinnings of (1) the decline of mobility function in PD, and (2) the effects of an engaging, cognitive game-based DT-walking program on mobility functions.

The first objective is to determine if the metabolic brain activity during single and DT treadmill walking is different in PD as compared to able-bodied age-matched controls. It is hypothesized that there will be significant changes in brain metabolic activity of several cortical and subcortical regions of the PD patients relative to able-bodied controls. This will be the case for both single and DT-walking conditions.

The second objective is to conduct a multi-centered, single-blind, randomized, two-arm, and parallel group-controlled trial and examine the effects of a 10-week DT treadmill training program: on (a) gate and cognitive function tested under single and DT conditions, (b) the pattern of metabolic brain activity quantified with single and DT-walking uptake periods, and (c) structural and functional changes (at rest) occurring prior to and after DT-walking training.

### Participants

52 patients with PD [26 from Winnipeg, MB, Canada (site 1) and 26 from Toronto, ON, Canada (site 2)], and 15 age-matched healthy, able-bodied control subjects (site 1) will be recruited in a 2-year time period. The criteria used for recruitment of the patients will include; (a) diagnosis of PD according to United Kingdom brain bank criteria (Hughes et al., 1992), (b) stage II-III of PD according to the Hoehn and Yahr scale (Bhidayasiri and Tarsy, 2012), (c) age 50–75 years, (d) adequate vision to see required images on a standard computer monitor, (e) able to score 26 or greater on the Montréal cognitive assessment scale [MoCA (Marinus et al., 2011)], (f) on stable medications for the past 3 months, and (g) able to walk overground for 2 min without an aid or cane. The exclusion criteria for participation will include the following: (a) diagnosis of any psychiatric comorbidity or history of any other neurological disease other than PD, (b) any other medical condition limiting participant ability to walk on the treadmill, and (c) neuroimaging contraindications (i.e., claustrophobia, severe dyskinesia, diabetes, non-compatible metal implants, etc.). Healthy able-bodied adults with the same inclusion/exclusion criteria listed above except for diagnosis of PD will be recruited.

All responders to calls for participation will undergo a screening assessment. Ethics approval has been obtained from the institutional Human Research Ethics Boards. All volunteer participants will be asked to read and sign the informed consent form. The Clinical trial has been registered at ClinicalTrials.gov, identifier: NCT04415775.

Successfully screened PD participants will be randomized to the experimental intervention (XG) or an active control arm (CG). A computerized block randomization procedure will be implemented by an independent statistician. Randomization will be stratified by stage of the disease (Hoehn and Yahr stage II and III). To ensure similar representation of men and women, randomization will also be stratified by sex. Randomization will be achieved by having participants choose an opaque sealed envelope, each containing a group assignment.

### Test Procedures and Protocols

#### Intervention Protocol

**FIGURE 1:**
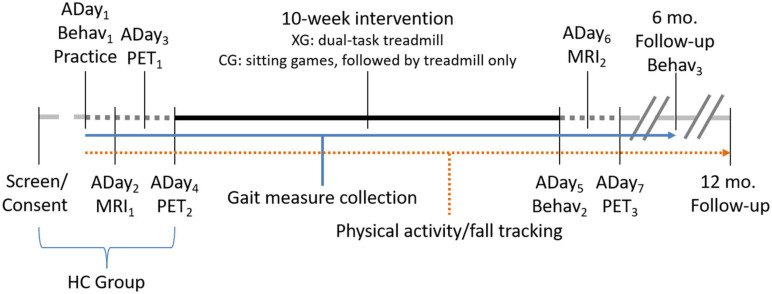
Experimental timeline. Assessment days (ADay) indicate order of assessments. PET scans on ADay_3_ and ADay_4_ will be counterbalanced between a single-task treadmill walking only task and a DT treadmill walking and cognitive game condition. A final DT PET scan will be acquired following intervention (ADay_7_). MRI scans will be collected pre- (ADay_2_) and post-intervention (ADay_6_). Behavioral testing (Behav; including motor measures and neuropsychological testing) will occur on three occasions: prior to intervention (ADay_1_), following intervention (ADay_5_), and again after a 6-month follow-up. Fall-history and physical activity tracking will be acquired throughout, until 12-months post-intervention. Measures of gait will be acquired throughout the program, including during behavioral assessment days, at each training session, and on PET assessment days. The experimental (XG) and control arm (CG) groups will complete the entire timeline, while healthy controls (HC) will complete up until ADay_4_.

The experimental timeline is visualized in Figure 1. All PD participants will undergo a 10-week exercise program, three times per week. Each therapy session will involve: (a) 10-min warm-up of balance exercises while standing on a sponge or balance disk, and (b) treadmill-walking of 3- to 5-min intervals and with 1-min rest periods for a total duration of 35 min. The type of compliant surface used, and the treadmill speed will be adjusted so that participants will not require the use of the handrails or any other body support. As with the gait assessments participants will be fitted with a body harness connected to a LiteGait support structure.

Participants in the CG group will complete 10 weeks of a “single-task” exercise program including videogames only (i.e., for 10 min while sitting) and walking-only on the treadmill—these tasks are conducted independently, one at a time. Participants in the XG will perform various cognitive computer games during the warm up period on the compliant surface and during all treadmill walking intervals. The computer games will be played using the head mounted IB-mouse as described above. Many inexpensive and easily accessible common and modern games exist which are visually rich, fun, and engaging. They include a variety of tasks that require; (i) visual search and tracking of multiple targets, (ii) speed accuracy requirements, (iii) presence of distracters, (iv) matching tasks, and (v) working memory. Note that the games that will be used during behavioral and PET assessments and those used in the XG exercise program are different.

As in most physical therapy programs, the specifics of DT training are customized for each patient, and the program gradually becomes more challenging as a patient’s performance improves. In the first DT training meeting, 6–10 games will be selected for each participant for their 10-week training session. They will be selected according to the domains of executive function, and also the personal preferences of the participant. Each game has hundreds of difficulty levels and as each level is successfully completed then the game automatically advances to the next difficulty level. Some games are less difficult than others, i.e., starting difficulty level can be easy, moderate, or hard. Thus, different games can be chosen to progress the difficulty level of the mental activities, i.e., speed of play, precision level, movement amplitudes, and distractions. Physical demands (i.e., type, thickness, and density of the compliant surface and the treadmill speed) will be progressed as tolerated. Participants in the CG group will be asked to play the computer video games using the IB-mouse while sitting for 10 min prior to treadmill walking. This will allow the CG group to have adequate practice using head rotation to control the IB-mouse which is used for assessment of DT performance.

#### Behavioral Testing

Behavioral testing includes motor and gait assessment and neuropsychological testing (described below). Behavioral testing will occur prior to intervention at baseline, immediately following intervention, and at a 6-month follow-up (see Figure 1). Gait measurements will be conducted throughout the duration of the experiment to track participant progress; however, it is important to note that these gait variables are in addition to those gathered under the more stringent assessment sessions. Additionally, physical activity tracking will take place continuously and will be acquired for 12 months following treatment.

##### Motor and Gait Assessment

Behavioral assessment of each PD participant will be conducted on three occasions, before and after a 10-week exercise program, and at a 6-month follow-up time period. Healthy control participants will be assessed at time 1 only. PD participants will initially be assessed with the Part-III (motor) section of the Movement Disorder Society—Unified Parkinson Disease Rating Scale [MDS-UPDRS (Goetz et al., 2008)]. Assessment of gait function under single-task (walk only) and DT conditions will be conducted on a treadmill, instrumented with a pressure mapping system. Treadmill walking is used to obtain 40–60 consecutive steps and at a constant speed [see Bhatt (2018) and Szturm et al. (2017) for a detailed description of data recording, analysis, and test-retest reliability]. Participants will be instructed to walk on the treadmill at their preferred gait speed without hand support for four 1-min intervals (one single-task walking only, one VMT, and two VCG trials, described below). The center-of-pressure of foot displacement in the mediolateral (ML) and anteroposterior (AP) directions will be collected at 100 Hz.

**FIGURE 2:**
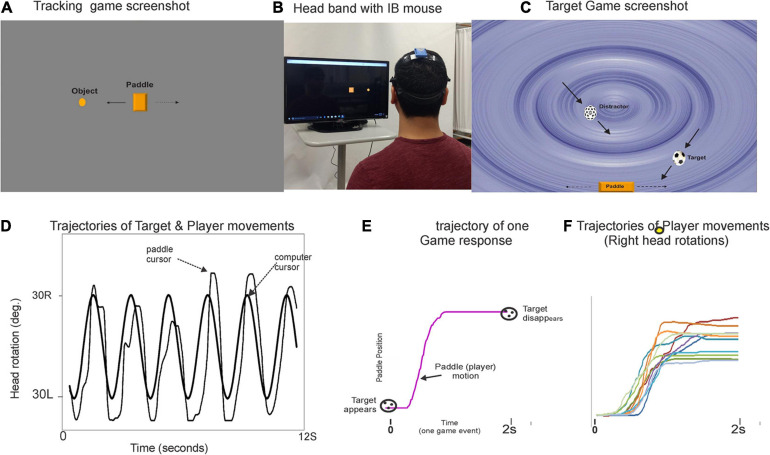
Illustration of the DT gaming set-up and snapshots of the visuomotor tracking (VMT, panel **A**) and visuospatial cognitive games (VCG, panel **C**). Panel **(B)** shows a participant wearing a plastic headband with inertial motion mouse. The goal of the VMT game is to track and overlap the game paddle (rectangle object) with a moving circle object (computer controlled). The goal of the VCG is to move the game paddle, catch target objects (soccer ball), and avoid distractors (dotted sphere). Panel **(D)** plot shows typical movement trajectory of a healthy young adult playing the game. Panel **(E)** presents a single game movement trajectory (game paddle coordinates) of one game event from target appearance (time zero) to target disappearance (time 2s). All segmented game movement trajectories of one game session are sorted and grouped by direction. Panel **(F)** presents overlay plots of the segmented and sorted game movement trajectories for a 60 s game trial. Presented are the game movement trajectories for rightward head rotations. Details on how to quantitate behavioral output measures have been published elsewhere (Nankar et al., 2017; Szturm et al., 2017; Ahmadi et al., 2019).

A custom computer application with the following two assessment modules will be used for the DT test conditions: (a) a visuomotor tracking (VMT) task and (b) a visuospatial cognitive game (VCG) task. Figure 2 illustrates the gaming set-up for the VMT and VCG tasks. As described previously (Nankar et al., 2017; Szturm et al., 2017; Ahmadi et al., 2019; Mahana et al., 2021), an IB-mouse will be used to control and interact with the cognitive assessment games. The VMT task used in the present study requires real-time, on-line visual feedback of the relative positions of two objects. The VCG task requires visual search to locate target objects and cognitive inhibition to avoid distractors. The VMT and VCG tasks require precision head-pointing movement. Many real-life tasks involve head movements to track and locate various objects, and for information processing of what are being seen. The increased visuospatial processing necessary to maintain walking rhythm and to correct for any drifting on the treadmill would compete for resources required to perform the VMT and VCG tasks, and vice versa.

A treadmill was chosen for the DT gait and cognitive functions for the following reasons. First, treadmill walking was chosen to overcome the confounding effect of gait speed. The treadmill allows the speed to be kept constant between walking conditions (single-task and DT), and over repeated measures (pre- and post-intervention time periods). This is of particular importance to standardize the uptake period of the PET imaging analysis at the three test periods. Most DT gait studies are performed overground, and all overground studies report a significant decrease in gait speed during the DT-walking condition, as compared to walk-only trials (Stegemöller et al., 2014; Raffegeau et al., 2019). Therefore, during overground DT-walking trials, there is a planned strategy to reduce the physical demands or threat to balance. Besides gait speed, many DT gait studies examine how cognitive demands affect gait rhythm and stability [i.e., recording spatiotemporal gait variables or analysis of trunk linear acceleration (Ijmker and Lamoth, 2012; Montero-Odasso et al., 2012; Asai et al., 2013; Martinez-Martin et al., 2013; Nankar et al., 2017; Ahmadi et al., 2019)]. However, gait speed is a confounding variable, as spatiotemporal gait variables and trunk acceleration are sensitive to changes in gait speed (Frenkel-Toledo et al., 2005; Jordan et al., 2007; Kang and Dingwell, 2008; Keene et al., 2016; Cole et al., 2017). Most overground DT-walking studies use an instrumented walkway, which records only 4–6 consecutive steps. It has been shown that using a continuous walking protocol, instead of short intermittent walks, and collecting more than 30 consecutive steps improved reliability, in particular for measures of gait variability and during DT-walking trials (Bruijn et al., 2009; Hollman et al., 2010; Galna et al., 2013). In addition, there is a limited choice of executive cognitive tasks that can be assessed during the short time period to walk a few meters. For example, cognitive tasks used during overground walking include walking while talking, recall of names/words, serial subtraction, or auditory Stroop (Stegemöller et al., 2014; Gregory et al., 2017; Ghanavati et al., 2018; Raffegeau et al., 2019; Snir et al., 2019). The present study extends these assessments to include visuomotor and visuospatial cognitive tasks assessed over a 1-min time period. Tracking visual objects and processing of what is being seen are important cognitive functions to consider in the analysis of DT interference on mobility (Nagamatsu et al., 2009; Logan et al., 2010). Cortical areas devoted to visual spatial ability such as the frontal and parietal lobes may be particularly susceptible to the neurodegenerative process of PD (Battisto et al., 2018; Lally et al., 2020). Following the principle of neural overlap (Crockett et al., 2017), DT interference should be greatest when the cognitive and motor tasks engage the same neural circuits and processing resources, e.g., visual-spatial processing. Walking endurance will be evaluated with a 6-min walk test [6MWT (Combs et al., 2014)]. Overground walking speed will also be evaluated over 25 m in a straight corridor.

##### Neuropsychological Tests

Parkinson’s disease has been associated with cognitive impairment across a range of domains, including aspects of cognitive flexibility, inhibitory control, sustained attention, and working memory (Owen et al., 1993; Uekermann et al., 2003; Kudlicka et al., 2011; Weintraub et al., 2018). The Cambridge Neuropsychological Test Automated Battery (CANTAB) for PD will be used for assessment of cognitive function. These will include; (a) Paired Associates Learning, (b) Pattern Recognition Memory, (c) One Touch Stockings of Cambridge, (d) Spatial Working Memory, and (e) Match to Sample Visual Search. Health-related quality of life will be assessed with the PD Questionnaire (Damiano et al., 1999). This neuropsychological data will be collected prior to participation commencement, at the end of the 10-week intervention, and at a 6-month follow-up, in the same session as the other behavioral assessments.

Fall rate will be recorded for a period of 1-year after completion of the exercise program. Falls rates will be considered a secondary outcome, due to concerns about the reliability and validity of self-report falls diary data. A fall will be defined as an unexpected event in which the participant comes to rest on the ground, or the floor (Mirelman et al., 2016). Several options will be used for the recording of fall events and to maximize the accuracy of reporting. Participants will receive fall calendars, either a paper version, a web-based calendar, or a smartphone application. Information logged in the online or smartphone-based calendars will automatically be uploaded to a database. Participants will be provided pre-addressed envelopes, and will be instructed to mail the paper version once every month. If a fall is recorded, the participant will be contacted by their treating therapist and asked when and how the fall occurred and whether there was any injury or need for medical treatment.

##### Physical Activity Tracking

In the second week of the exercise, program participants will be given a Fitbit watch (Ionic) to track physical activity levels during waking hours (Liang et al., 2018; Tedesco et al., 2019; Fuller et al., 2020). This model records steps taken, GPS and heart rate. Participants will be shown how to use the watch and how to access and transfer the stored data. Recorded activity data from the watch will be transferred to the therapist once a week during a treatment session. Over a 10-week time period this will provide ample practice on how to use the Fitbit watch and how to access and transfer its recorded activity data. Following the 10-week exercise program, the participants will be shown how to transfer their activity data to the research team. They will be instructed to do this once a week. Participants will be sent an e-mail or text message to remind them to do so.

Walks of more than 1 min will be determined by data showing continuous steps and where there are concurrent and adequate changes in GPS and increased heart rate. For each walking bout exceeding 1 min, the walking distance will be determined using the GPS data. The main physical activity outcome measures will be the total monthly walking duration and walking distance. Walking speed will also be computed for each walking bout over 1 min, and a monthly average obtained. Following the exercise program, all participants will be contacted by phone once a month to remind them of the use of the physical activity tracker.

#### Image Acquisition

##### PET Data

All subjects will undergo two pre-intervention [^18^F] FDG-PET scans after fasting for at least 6 h before scanning. A head CT scan will be acquired for attenuation correction purposes. Static images (10 min) will be acquired starting 40 min after injection. During the FDG uptake period, participants will perform in different sessions, on different assessment days; (a) treadmill walking only (test session 1) and (b) DT treadmill walking (test session 2; see Figure 1). The order of the walk-only and DT-walking sessions will be counterbalanced. The single and DT treadmill walking will be done for 28 min each. This will involve seven 3-min walk intervals with 1-min rest periods (standing). Walking speed and duration will be carefully controlled, and repeated for each test session (i.e., single and DT conditions, pre- and post- intervention). Seven different standardized cognitive games will be selected for the seven DT-walking intervals. These will include; (a) five 3-min VC games with different difficulty levels (i.e., increasing game speed, adding distractors, and changing path of target motions), and (b) two 3-min VMT games, one played using horizontal head rotations and one with vertical head rotations. The PD patients will repeat the DT-walking PET imaging protocol after completion of the 10-week exercise program.

##### MRI Data

All participants will undergo an MRI session before the intervention, Data collected will include a structural, T_1_-weighted anatomical image, a T_2_^∗^-weighted resting state fMRI scan, pCASL, and DTI data. Following the intervention, all PD participants will undergo a second MRI session, including the same scans as those acquired before intervention.

First, structural T_1_-weighted (T1w) MR images will be acquired, using a 12-channel head and neck coil. T1w data will be acquired using an MP-RAGE sequence with TR/TE/TI = 2300/3.02/900 ms, 240 slices, flip angle = 9, FOV = 256 mm × 256 mm, 1.0 mm^3^ (isotropic) resolution. This T1w data will provide the anatomical data over which the PET, RS-fMRI, pCASL, and DTI will be overlaid. Although MRI data cannot be used to directly investigate neural changes occurring during DT-walking, this technology will provide valuable information regarding how brain function and structure may change before and after DT-training in PD participants.

RS-fMRI data (T_2_^∗^-weighted) will be collected using a gradient-echo, echo planar imaging (GE-EPI) sequence with the following parameters: TR/TE = 2000/28 ms, 240 slices, flip angle = 77, FOV = 220 mm × 220 mm^2^, 3.4 mm × 3.4 mm × 4.0 mm resolution. The parameters for the resting state pCASL data are as follows: TR/TE = 4000/12, 20 slices, flip angle = 90, FOV = 240 mm × 240 mm^2^, 3.8 mm × 3.8 mm × 5.0 mm resolution, inter-slice space = 1 mm, labeling time = 2.0 s, post label delay time = 1.2 s, bandwidth = 3 kHZ/pixel. For each participant, 45 label/control image pairs will be acquired. DTI data will be acquired using a single-shot spin echo, echo planar imaging sequence with the following parameters: 35 directions (*b* = 700 s/mm^2^), TR/TE = 10413/70.8 ms, FOV = 240 mm × 240 mm × 163 mm, 65 transverse slices, 2.5 mm^3^ (isotropic) resolution.

### Data Analysis

Details of the analysis procedures used to quantify the following spatio-temporal gait variables, and the VMT and VCG performance measures have been published elsewhere (Nankar et al., 2017; Szturm et al., 2017; Ahmadi et al., 2019). In brief, the following spatio-temporal gait variables were computed from the center-of-pressure of foot displacement time series recordings; (a) step length (SL), the distance between two successive heel contacts in the AP direction, and (b) swing time (SW) time, the time between toe-off and heel contact of each leg. The average SL and SW were then determined for each 1-min walk trial. Variability measures quantify the inter-stride fluctuations in SL and SW around the mean value and will be report it as coefficient of variance, COV-SL and COV-SW. The location of all heel contacts during each 1-min walk trial were also determined in both AP and ML directions. The dispersion of heel contact locations will be computed as the COV, and reported as AP-drift and ML-drift.

Figure 2 illustrates the VMT and VCG game tasks and plots of game movement responses. The following outcome measures for the VMT task include: (a) total residual error (TRE), determined by computing the difference between the trajectories of the target and head cursor motions, (b) amplitude variability (AV), a measure of movement consistency determined as the coefficient of variation of the mean right-to-left and left-to-right tracking movements for 20 cycles (i.e., a 45 s. trial). The following performance measures for the VCG will be quantified: (a) success rate (SR), (b) response time (RT), and (c) movement variance (MV).

#### Statistical Analyses

Our sample size was calculated based on our previous work involving PET imaging in PD in which participants were randomized into either gene therapy or sham-surgery groups, and assessed at 6 months follow-up (Ko et al., 2014a). With anticipated ρ2 = 0.673 [20% more than what is expected for placebo effect-related pattern’s explanation of clinical benefits induced by sham surgery over 6-months (Ko et al., 2014a)], G^∗^Power (version 3.1.9.2; RRID:SCR_013726) estimates the minimal sample size to be 12 (effect size f2 = 2.060). With maximally anticipated drop-out rate of 10% (e.g., motion artifacts, patient’s voluntary withdrawal, etc.); we require 13 subjects per group/site.

MATLAB (The Math Works Inc., Natick, MA, United States version 2010a) will be used to compute the outcome measures. Normality of the data will be assessed using the Shapiro-Wilks test. Sphericity will be assessed using Mauchly’s test. For normally distributed data sets, a mixed-model ANOVA will be used to examine the effects of time (pre– and post-intervention), group (XG and CG) and interaction of time^∗^group of all behavioral outcome measures (gait, cognitive, neuropsychological, fall rates, physical activity levels, and health-related quality of life). Note the time factor for fall rates, physical activity outcome measures, and health-related quality of life will include the 6-month and 12-month follow-up time periods. Non-normally distributed data will be assessed using the Wilcoxon-Signed rank test to determine differences between pre- and post-intervention. The significance level will be set at *p* < 0.05, and statistical analysis will be conducted using SPSS (v.22; SPSS Science, Chicago, IL, United States).

For the neuroimaging data, Aim 1 will use PET data to compare levels of metabolic activity by region between; (a) single-walk only and DT-walk trials, and (b) PD and able-bodied age-matched controls. Aim 2 will use PET and MRI (RS-fMRI, pCASL, and DTI) data to investigate neural changes (whole-brain and ROIs) between pre- and post-intervention. See Table 1 for the ROIs to be used in the PET and RS-fMRI data for Aims 1 and 2. Paired sample *t*-tests will be conducted for these different neuroimaging modalities, with age and gender included as regressors of no interest. Additional correlational analyses are planned to investigate the relationship between changes in neural activity and behavioral metrics such as cognitive task performance and gait measures.

**TABLE 1:** Regions of interest for the PET and fMRI ROI analyses.

**Amygdala, L**	**Dorsolateral PFC, L**	**Inferior Temporal, R**	**Mesial Temporal, L**	**Orbital frontal, R**	**Thalamus, R**
Amygdala, R	Dorsolateral PFC, R	Insula, L	Mesial Temporal, R	Putamen, L	Uncus, L
Anterior Cingulate, L	Frontal pole, L	Insula, R	Midbrain	Putamen, R	Uncus, R
Anterior Cingulate, R	Frontal pole, R	Lateral Temporal, L	Middle Temporal, L	Substantia nigra	Ventral Striatum, L
Subgenual cingulate (BA 25)	Hippocampus, L	Lateral Temporal, R	Middle Temporal, R	Superior Temporal, L	Ventral Striatum, R
Caudate, L	Hippocampus, R	Medial PFC, L	Occipital	Superior Temporal, R	Ventrolateral PFC, L
Caudate, R	Inferior Temporal, L	Medial PFC, R	Orbital frontal, L	Thalamus, L	Ventrolateral PFC, R

*L, left; R, right; PFC, prefrontal cortex.*

#### Imaging Analyses

##### Preprocessing

Neuroimaging data will be converted from dicom to NIfTI format using the dicm2nii converter, and will then be preprocessed using different software toolboxes, largely based in SPM12 (^1^ RRID:SCR_007037), in MATLAB^®^ (2017b; The MathWorks Inc., Natick, MA, United States). The RS-fMRI data will be preprocessed and analyzed using the functional connectivity toolbox (CONN;^2^ RRID:SCR_009550). The pCASL data will be preprocessed and analyzed in ASL Perfusion MRI data processing toolbox (^3^ RRID:SCR_005997). Functional data will undergo motion correction, coregistration with the structural (T1w) image, segmentation/spatial normalization, and spatial smoothing (8 mm full-width at half maximum Gaussian kernel). Segmentation/spatial normalization for T1w data will use DARTEL registration to the MNI152 template, using the CAT12 toolbox (^4^ RRID:SCR_019184). During the segmentation, white matter, gray matter and cerebrospinal fluid will be isolated; WM will be masked out from the functional analyses. Normalized images will be resliced to 1.5 mm (isotropic). The resulting deformation field will be applied to the functional images.

##### FDG-PET Analysis

For FDG-PET data, an in-house whole-brain mask will be used as the explicit mask. PET images will be proportionally scaled to each image’s global values for statistical analysis. For both walk only and DT-walking conditions FDG-PET, ordinal trend/canonical variate analysis [OrT/CVA (Habeck et al., 2005)] will be applied to characterize the normal DT-walking-related brain metabolic pattern (NDWP), by comparing FDG-PET of single-walking vs. DT-walking in the healthy controls. First, the OrT transforms the preprocessed image data to increase the salience of ordinal trend effects, allowing the proceeding principal component analysis (PCA) to identify principal components with targeted expression (i.e., a consistent increase of pattern expression at the individual subject level), while also controlling for the salience of untargeted expression (Habeck et al., 2005). The principal components that result from the OrT can either be used individually, or can be linearly combined, allowing the expression of a given metabolic pattern to be quantified in each scan. Similarly, the PD-specific DT-walking-related brain metabolic pattern (PDWP) will be produced (i.e., DT-walking vs. single-walking in PD patients at pre-intervention). Additionally, we will examine if there is a different metabolic pattern, specific to the 10-week treatment-induced clinical benefits in PD by OrT/CVA (post- vs. pre-intervention during DT-walking). The topographical rating scale of the proposed FDG-PET patterns (i.e., NDWP, PDWP, and longitudinal pattern) will be computed in each FDG-PET image. Furthermore, behavioral scores (such as gait and cognitive performance) will be correlated with changes in metabolic patterns from pre- to post-intervention. The topography of the resulting spatial patterns will be compared using similarity test correcting for auto-correlation (Ko et al., 2014b).

##### RS-fMRI Analyses

RS-fMRI will undergo two types of analyses in the CONN toolbox: (1) ROI-to-ROI analyses (using the ROIs listed in Table 1), and (2) a whole-brain independent component analysis (ICA). For the ROI analyses, region-to-region connectivity (z-matrix) will be extracted using the 210 V Maximum Probability Map parcellation method (Glasser et al., 2016). The z-matrix will be sorted and undirected adjacency matrices will be defined with varying cost (1–50%). For example, at 25% cost threshold, the top 25% of the z-values will be set to 1 and the rest set to 0, excluding the diagonal elements. Global graph theory metrics (e.g., characteristic path length, clustering coefficient, smallworldness, global efficiency and mean local efficiency) will be compared between groups (Watts and Strogatz, 1998; Humphries and Gurney, 2008; Rubinov and Sporns, 2010). Degree centrality (DC) refers to the number of regions that are functionally connected to a region—this is an appropriate connectivity metric for the dlPFC in PD as this region is important in top-down cognitive control. Betweenness centrality (BC) refers to the number of times a region (i.e., node) is along a shortest path between region-pairs and thus is a suitable connectivity metric to assess convergent neuroanatomical structure, such as the caudate nucleus (Alexander et al., 1986). Both DC and BC will be computed at a regional level. Recently, we demonstrated that DC of the left dlPFC correlates with inhibition control performance in normal healthy individuals (unpublished pilot data) while the BC level of the caudate correlates with cognitive performance in non-demented PD patients (Wright et al., 2020).

For the whole-brain analyses, data will be run through an ICA in CONN, which separates the RS-fMRI data into components, with the signal of these components being maximally independent from each other (Calhoun et al., 2009). The components separated using ICA tend to separate into groups, comprising highly replicable resting state networks: regions whose activity is correlated over time, in the absence of a task. Several resting state networks of interest will be investigated in pre- vs. post-intervention, including the central executive, sensorimotor, visual, default mode, and cerebellar networks, as these networks are related conceptually to the motor and cognitive deficits observed in PD and many have been shown to be altered in PD in the past (de Schipper et al., 2018; Wolters et al., 2019; Dong et al., 2020; Schneider et al., 2020).

##### pCASL Analysis

For the pCASL images, the preprocessed cerebral blood flow (CBF) images will be assessed using SVM-ISDA, which will be used to train a classifier that separates patients into PD patients with normal cognition, and those with mild cognitive impairment. The pCASL data will be analyzed by Machine-based Alzheimer’s disease Designation (MAD) to compute Alzheimer’s-like pattern expression scores (Katako et al., 2018). High scores in metabolic/perfusion patterns indicate similarity with Alzheimer’s patients—we have previously shown that PD dementia patients show similar brain metabolic patterns and that perfusion imaging may replace FDG-PET imaging when MAD is applied (Katako et al., 2018). Our recent work suggests the utility of using SVM-ISDA-based scores for monitoring disease progression in repeated measures designs. Changes in these scores will be used to assess the neural correlates of the treatment response.

##### DTI Analysis

Diffusion tensor imaging data will be preprocessed using SPM12 and the artifact correction in diffusion MRI toolbox (ACID;^5^ RRID:SCR_010470)) and after coregistration of each participant’s diffusion weighted data with their T1w image, data will undergo eddy current and motion correction, before fitting the diffusion tensor, using an ordinary least squares algorithm. CAT12 will be used for segmentation and skull stripping of the b0 map. Fiber tracking, fractional anisotropy (FA), axial diffusivity (AD), radial diffusivity (RD), and mean diffusivity (MD) map creation will be done using the ACID toolbox. ROIs [e.g., forceps minor, corpus callosum, uncinate fasciculus, and cingulum (Ghanavati et al., 2018; Snir et al., 2019)] will be investigated.

## Anticipated and Preliminary Results

The anticipated results of this study can be broken down by objective. First, in comparing if and how metabolic brain activity differs during single and DT-walking in PD patients, compared to able-bodied controls, we will likely observe increased activation of sensorimotor and cerebellar brain regions and decreased activation in the PFC of the PD patients. We hypothesize that these changes in brain activation patterns will be associated with decreased gait and cognitive performance measures and the magnitude of DT effects. Second, we hypothesize that both training programs will result in significant improvements in gate and cognitive function with medium to large effect sizes, and that the DT training program will result in significantly greater improvements in the XG compared to CG, also with medium to large effect sizes. It is hypothesized that the DT treadmill-training program will result in specific and significant changes of the DT gait-related abnormal brain metabolic pattern observed in Objective 1.

Our behavioral and PET hypotheses are supported by a recent feasibility study that we completed in which 15 PD patients (Hoehn and Yahr stages II-III) participated in a 10-week DT-treadmill gait training program using the VMT and VCG as described in the “Methods” section above (Mahana et al., 2021). Significant improvements in the majority of gait variables (average and COV of SL and SW) with medium to large effect sizes were observed following the intervention. This was the case for both walk-only and DT-walking assessments. There were also significant improvements in VMT and VCG outcome measures post-intervention with medium to large effect sizes. This was the case when tested while sitting and while treadmill walking. In addition, a qualitative analysis of the participant’s experiences revealed that the program was challenging and required a high degree of mental processing. The majority of participants reported that the games were engaging and they appreciated the variety of games used for the DT training. Also, to note 3 of the 15 participants did not like playing the games.

In a recent pilot study, we tested the feasibility of imaging the DT-walking-related changes in brain glucose metabolism in patients with PD (Szturm et al., 2020). Fifteen patients with PD (stage II and III) were scanned with FDG-PET. Ten patients were rested during the FDG uptake period and five patients performed DT treadmill walking for 28 min. All five PD patients receiving the DT-walking protocol showed a consistent DT interference of gait measures (average and COV) and of all VMT and VCG performance measures. Glucose metabolism was significantly increased in several brain regions of the patients who performed the DT treadmill walking as compared to those who rested during the uptake period. These regions include primary visual/sensorimotor areas, thalamus, superior colliculus, and cerebellum. When individual results were analyzed, patients in the earlier stage of PD (Hoehn and Yahr stage II) showed increased FDG uptake in the PFC regions during DT treadmill walking as compared to resting levels. More progressed PD patients (Hoehn and Yahr stage III) did not show any notable increase in PFC activity during DT treadmill walking. It should be also noted that patients with mild cognitive impairment (MCI) also show suppressed PFC activity during walking (Holtzer and Izzetoglu, 2020).

In regards to the pilot MRI data, we expect significant neural changes across modalities. Due to the importance of the dorsolateral PFC for both cognition and gait, we expect both PD groups to have reduced functional connectivity between this region and other basal structures compared to able-bodied controls using RS-fMRI data (Monchi et al., 2004; Nagano-Saito et al., 2014). We expect that following intervention, the XG group will have increased functional connectivity between these regions compared to the CG group, and that this functional connectivity will correlate with PD stage. Similarly, in the whole-brain RS-fMRI analysis, we hypothesize that pre-intervention, both PD groups will have altered functional connectivity within central executive, sensorimotor, visual, default mode, and cerebellar resting state networks compared to healthy controls (de Schipper et al., 2018; Wolters et al., 2019; Dong et al., 2020; Schneider et al., 2020), but that PD groups will not differ from each other. Post-intervention, we hypothesize that functional connectivity within these networks will differ between XG and CG PD groups. For the pilot pCASL data, we hypothesize that SVM-ISDA scores will not differ between PD groups at baseline, but will differ from able-bodied controls. Following intervention, we hypothesize that scores for both XG and CG groups will be reduced, with a larger effect observed for the XG group, indicating a larger treatment response. Although the DTI data are largely pilot data in nature, based on previous research indicating that reduced white matter integrity in forceps minor, corpus callosum, uncinate fasciculus, and cingulum is associated with reduced gait stability and may be associated with an increased risk of falling (Ghanavati et al., 2018; Snir et al., 2019), we hypothesize that the same relationship will hold in our sample. While it is thus far unclear whether structural changes in DTI can be observed in our population following 10-weeks of intervention, we expect that increased white matter integrity at post-intervention will be associated with improved gait stability and reduced number of falls at the 6-month follow-up.

## Discussion

An integrated approach to treatment with embedded assessment of balance, gait, visuomotor, and cognition has the potential to impact both the prevention and rehabilitation of mobility limitations, and in cognitive function. Improved mobility functioning in PD directly translates to improved community ambulation, as well as increased physical activity and social participation. These benefits are known to have a significant preventative and disease-modifying impact.

New telerehabilitation technologies will likely improve clinical outcomes by making therapy more available, more motivating, and more specific and effective. While one of the pitfalls of the present study includes possible patient attrition, previous pilot-testing work showed high retention of participants, partly due to the engaging nature and low-level of adverse events related to the intervention (Mahana et al., 2021). The computerized DT platform presented in this study broadens the type of standardized visuomotor and executive cognitive activities for use for DT balance-gait training that has previously been reported. A comprehensive analysis of spatial and temporal features of steady state gait has a greater validity to measure gait performance (rhythm, pacing, and stability) as compared to gait speed alone. The use of interactive digital media provides a flexible method to produce a wide range of executive cognitive activities while performing complex motor behaviors such as walking. The types and amount of cognitive stimulation participants engage in during intervention needs to be objectively measured, and this will help clarify the potential added benefit of activities beyond physical exercise alone. Quantification of cognitive-motor interactions has the potential to be a valid non-invasive biomarker for early detection of balance-mobility limitations and cognitive decline in the early stage of disease, and with aging, although these results will not be generalizable to later stages of PD. Although participant motion is a potential pitfall for the neuroimaging data, only early-stage PD patients with controlled tremor will be recruited. Outcomes of this research will provide new insights into brain plasticity mechanisms and will accelerate further optimization and commercialization of multi-modal mobility-cognitive training applications along with accompanying smart electronic monitoring tools.

The computer game-based treadmill platform and embedded automated electronic monitoring tools were designed to transition from physiotherapy clinics to community fitness/wellness centers, as well as assisted-living complexes. Improved and cost-effective methods of screening and fall risk assessment in the community, linked with highly-effective interventions and eHealth tools, will allow more individuals with PD to have access to, and the support of, personalized, scientifically motivated rehabilitation, no matter where they are located. In addition, the availability of electronic outcome measures and, ultimately, the creation of a web-based portal, will help optimize the platform and strengthen accountability.

While the focus of the present proposal is on the study of brain-behavior relationships and neuroplasticity mechanisms in PD, our DT mobility-training platform and behavioral PET and MRI brain imaging methods are directly applicable to other diseases that affect gait and cognition (e.g., cognitive vascular impairment, Alzheimer’s disease, and aging).

## Ethics Statement

This study has received ethical approval from the University of Manitoba’s Biomedical Research Ethics Board and this clinical trial has been registered at ClinicalTrials.gov, identifier: NCT04415775.

## Author Contributions

TS, TK, and JK: conceptualization and writing – original draft. TS, TK, BM, AG, DH, JM, AS, and JK: writing – review and editing. TS, AS, and JK: funding acquisition. All authors contributed to the article and approved the submitted version.

## Conflict of Interest

AS is a consultant for Hoffman La Roche and received honoraria from GE Health Care Canada Ltd., Hoffman La Roche. The remaining authors declare that the research was conducted in the absence of any commercial or financial relationships that could be construed as a potential conflict of interest.

## Footnotes

1 https://www.fil.ion.ucl.ac.uk/spm/software/spm

2 http://nitrc.org/projects/conn

3 https://www.cfn.upenn.edu/~zewang/ASLtbx.php

4 http://www.neuro.uni-jena.de/cat/

5 http://www.diffusiontools.com/
