# Supplementary material for: Changes in Metabolic Activity and Gait Function by Dual-Task Cognitive Game-Based Treadmill System in Parkinson’s Disease: Protocol of a Randomized Controlled Trial
Source: Front Aging Neurosci. 2021 Jun 4;13:680270. doi: 10.3389/fnagi.2021.680270 (PMC8211751; doi:10.3389/fnagi.2021.680270)
Supplement: Supplementary file 1 [file Table_1.DOCX]

**Supplementary Data**

Table S1: Commercial computer games purchased from on-line website [www.Bigfishgames.com](http://www.Bigfishgames.com). For matching and shooting games, participants use a small wireless hand-held clicker with a left mouse button to press when needed.

| **Big Fish Game** | **Axis Play** | **Start Difficulty** | **Type** | **Clicker** | **Precision** | **Background** | **Distractor** | **Executive Function** |
| --- | --- | --- | --- | --- | --- | --- | --- | --- |
| Abundante! | Horizontal | Difficult | Match 3 | Yes | Moderate | Low Optokinetic | No | Matching and Puzzle Solving |
| Action Ball | Horizontal | Moderate | Brick Buster | No | Moderate | High Optokinetic | Yes | Visual Tracking & Spatial |
| Acqua Ball | Horizontal | Easy | Brick Buster | No | Low | Medium Optokinetic | Yes | Visual Tracking & Spatial |
| Astrobugs Revenge | Horizontal | Difficult | Match 3 | Yes | High | Medium Optokinetic | No | Matching |
| Ark Light | Variable | Moderate | Shooting | Yes | Moderate | Medium Optokinetic | Yes | Search and Select |
| Birds Town | Horizontal | Moderate | Match 3 | Yes | High | Low Optokinetic | No | Matching |
| Brave Piglet | Vertical | Moderate | Shooting | Yes | High | Low Optokinetic | Yes | Visual Tracking & Spatial |
| Bricks of Egypt | Horizontal | Easy | Brick Buster | No | Variable | Low Optokinetic | Yes | Visual Tracking & Spatial |
| Butterfly Escape | Horizontal | Moderate | Match 3 | Yes | High | Low Optokinetic | Yes | Visual Tracking & Spatial |
| Chicken Invaders | Variable | High | Shooting | Yes | Moderate | High Optokinetic | Yes | Search and Select |
| Clear It 2 | Variable | High | Match 3 | Yes | High | Moderate Optokinetic | No | Visual Tracking & Spatial |
| Digby Donuts | Horizontal | Moderate | Catch &Sort | Yes | Moderate | Low Optokinetic | No | Search precision Sort |
| Dreamland Solitaire: | Horizontal | Easy-difficult | card sort | Yes | Low | Low Optokinetic | Yes | Matching Sorting |
| Egyptian Ball | Horizontal | Difficult | Brick Buster | No | Moderate | High Optokinetic | No | Search and Select |
| Gummy Drop | Horizontal | Moderate | Match 3 | Yes | High | Moderate Optokinetic | Yes | Match 3, Aligning |
| Hyperballoid 2 | Horizontal | Difficult | Brick Buster | No | Moderate | High Optokinetic | No | Visual Tracking & Spatial |
| Invadazoid | Horizontal | Difficult | Brick Buster | No | High | Moderate Optokinetic | Yes | Visual Tracking & Spatial |
| Jar of Marbles | Horizontal | Easy | Match 3 | Yes | Medium | Low Optokinetic | No | Matching Three , aligning |
| Jet Jumper | Variable | Difficult | Driving Game | Yes | High | High Optokinetic | Yes | Visual Tracking and Driving |
| Luxor 3 | Horizontal | Moderate | Match 3 | Yes | High | Moderate Optokinetic | Yes | Match 3, Aligning |
| Luxor HD | Horizontal | Moderate | Match 3 | Yes | High | Moderate Optokinetic | Yes | Match 3, Aligning |
| Pirates of Black Cove | Variable | Easy | Aim and Move | Yes | Moderate | Moderate Optokinetic | Yes | Search and Select |
| Plumboon | Vertical | Difficult | Match 3 | Yes | High | Moderate Optokinetic | No | Visual Tracking & Spatial |
| Reaxion | Horizontal | Moderate | Brick Buster | No | Variable | Moderate Optokinetic | Yes | Visual Tracking & Spatial |
| Ricochet Recharge | Horizontal | Moderate | Brick Buster | No | High | Moderate Optokinetic | No | Visual Tracking & Spatial |
